# Excitons and Phonons in Two-Dimensional Materials: From Fundamental to Applications

**DOI:** 10.3390/nano13233047

**Published:** 2023-11-29

**Authors:** Maciej R. Molas

**Affiliations:** Institute of Experimental Physics, Faculty of Physics, University of Warsaw, 02-093 Warsaw, Poland; maciej.molas@fuw.edu.pl

The isolation of graphene opened the gate to investigate a vast family of two-dimensional (2D) layered materials. The concept of an exciton, an electron–hole pair (e–h) bound by Coulomb interactions, is the foundation of solid-state physics. Excitons are responsible for the electronic and optical response of many semiconductors. Particularly, an optical response of semiconducting transition metal dichalcogenides (S-TMDs), e.g., MoS_2_, WS_2_, and WSe_2_, is dominated by the emergence of excitons even at room temperature [1]. Simultaneously, the lattice dynamics in solids are described by phonons [2]. They not only characterise the vibrations of atoms, but can also significantly influence the light–matter interaction due to electron–phonon or exciton–phonon (e–p) coupling [3]. Recently, a variety of intriguing excitonic complexes have been identified and described in monolayers of 2D materials: so-called bright and dark complexes, neutral and charged excitons, biexcitons, etc. [4]. A family of excitons is even larger in multilayered specimens and artificial van der Waals (vdW) heterostructures [5]. Phonons are also present in the optical response of different layered materials, apparent as phonon replicas. Consequently, the investigation of the phonon modes in 2D materials on account of e–p coupling is essential in terms of potential applications of layered materials.

The purpose of this Special Issue entitled “Excitons and Phonons in Two-Dimensional Materials: From Fundamental to Applications” is to provide a unique international forum and to cover the entire range of fundamental and applied research associated with excitonic complexes and phonon modes in two-dimensional layered materials. This Special Issue is composed of nine published papers [6,7,8,9,10,11,12,13,14] devoted to investigations of different 2D materials, such as S-TMDs, perovskites, and the multilayered structure of thin films, with theoretical [7,9,11,13] and experimental [10] approaches, as well as their combination [6,8,12,14].

The theory of excitons in atomically thin semiconductors was explored in Ref. [7] using a tight-binding model of the electronic structure. This comprehensive work includes an extensive review of the literature on 2D van der Waals materials, with a particular focus on their optical response from both experimental and theoretical points of view; this is followed by ab initio calculations of the electronic structure of MoS_2_, the construction of e-h pair excitations from the mean-field-level ground state, and a discussion of on-local screening of various geometries usually used in experiments. The paper concludes by adding another layer and showing the formation of excitons in heterostructures built from 2D semiconductors. The role of non-local screening in the exciton fine structures of WSe_2_ monolayers (MLs) is also theoretically analysed in detail in Ref. [14] in various dielectric-layered environments by solving the first-principles-based Bethe–Salpeter equation. The authors reveal that the influence of the dielectric environment on the exciton fine structures of S-TMD ML is surprisingly limited, pointing out that the non-locality of Coulomb screening plays a key role in suppressing the dielectric environment factor and drastically shrinking the fine structure splitting between bright and dark exciton states in the ML. The fine structure of the splitting of excitonic transition was also determined in (PEA)_2_PbI_4_ two-dimensional perovskites with the aid of polarisation-resolved optical spectroscopy in Ref. [10]. The phonon-assisted sideband characteristic for (PEA)_2_PbI_4_ was demonstrated to be split and linearly polarised, mimicking the characteristics of the corresponding zero-phonon lines. Interestingly, the splitting of differently polarised phonon-assisted transitions can be different from that of the zero-phonon lines. The ultrafast dynamics of valley-polarised excitons in monolayer WSe_2_ were examined using transient reflection spectroscopy in Ref. [12]. It was found that the anisotropic valley population of excitons decays on two different timescales. The shorter decay time of approximately 120 fs is related to the initial hot exciton relaxation related to the fast direct recombination of excitons from the radiative zone, while the slower picosecond dynamics correspond to valley depolarisation induced by Coulomb-exchange-driven transitions of excitons between two inequivalent valleys. The results, presented in Refs. [7,10,12,14], underline the variety and complexity of excitonic physics in layered 2D materials.

In the next level of studies of van der Waals materials, researchers moved onto studying compounds characterised by strong in-plane anisotropy, in contrast to S-TMDs with high in-plane symmetry. The optical response of bulk germanium sulphide (GeS) was systematically tested using different polarisation-resolved experimental techniques, such as photoluminescence (PL), reflectance contrast (RC), and Raman scattering (RS), as well as with the help of density functional theory (DFT) calculations in Ref. [6]. Both the RC and PL spectra are linearly polarised along the armchair direction, which confirms the in-plane anisotropy of GeS. The measured RS spectra over a broad range from 5 to 300 K consist of six Raman peaks, where polarisation orientations can be sensitive or not to the different excitation energies used. Research on the phonon modes is also described in Ref. [14], where a resonance RS study is performed in the domes of monolayer MoS_2_ using 23 different laser excitation energies covering the visible and near-infrared (NIR) ranges. The analysis of the intensities of the two first-order peaks, A′_1_ and E′, and the double resonance 2LA Raman band as a function of the laser excitation furnished the values of the energies of the indirect exciton and the direct excitonic transitions in the strained MoS_2_ domes. As shown in Refs. [6,14], the Raman scattering spectroscopy is a powerful tool to unveil the crystallographic structure of materials and to describe electron–phonon coupling.

Optical measurements under externally applied stresses or electric fields give the opportunity to study the electronic structure by comparing the evolution of optical peaks obtained from experiments and theoretical calculations. In Ref. [8], the stress-induced changes in electronic structure for the thermodynamically stable 1T polytype of selected MX_2_ compounds (M = Hf, Zr, Sn; X = S, Se) are determined using the density functional theory. The studied transitions are optically active and exhibit in-plane polarisation of light with negative pressure coefficients. Furthermore, the experimentally measured pressure coefficients of the HfS_2_ and HfSe_2_ absorption edges are in perfect agreement with the theoretical predictions. Multilayered van der Waals heterostructures based on transition metal dichalcogenides are suitable platforms on which to study interlayer (dipolar) excitons, in which electrons and holes are localised in different layers. A systematic analysis is carried out in Ref. [11] of the spin-valley physics in MoSe_2_/WSe_2_ heterobilayers under the influence of an external electric field and changes in interlayer separation. The study provides fundamental microscopic insights into the spin-valley physics of van der Waals heterostructures, which are relevant to understanding the valley Zeeman splitting of dipolar excitonic complexes, as well as intralayer exciton. The aforementioned results reveal an important role of external perturbations, such as pressure and electric field, in investigations of electronic and optical properties of layered 2D materials.

Ref. [9] is devoted to a numerical analysis of the designed absorber with high absorptivity and ultra-wideband (UWB), which ranged from the visible light range and near-infrared band using simulation software. The absorber, constructed using two-layer square cubes stacked on the four-layer continuous plane films, presented the properties of UWB and high absorptivity due to the combination of the anti-reflection effect and the three different resonances.

To conclude this overview of the papers published in the Special Issue entitled “Excitons and Phonons in Two-Dimensional Materials: From Fundamental to Applications”, I am confident that the readers will enjoy these contributions and may be able to find inspiration for their own research within this Special Issue. This series of manuscripts on this topic will give maximum impact and allow scientists to apply the same methodologies in understanding the mechanisms of physics in their systems.

## Data Availability

No new data were created or analyzed in this study. Data sharing is not applicable to this article.
